# The Cell Membrane of *Sulfolobus* spp.—Homeoviscous Adaption and Biotechnological Applications

**DOI:** 10.3390/ijms21113935

**Published:** 2020-05-30

**Authors:** Kerstin Rastädter, David J. Wurm, Oliver Spadiut, Julian Quehenberger

**Affiliations:** Research Division Biochemical Engineering, Faculty of Technical Chemistry, Institute of Chemical, Environmental and Bioscience Engineering, TU Wien, 1060 Vienna, Austria; kerstin.rastaedter@tuwien.ac.at (K.R.); david.wurm@tuwien.ac.at (D.J.W.); oliver.spadiut@tuwien.ac.at (O.S.)

**Keywords:** archaea, *Sulfolobus*, homeoviscous adaption, membrane, tetraether, diether, lipids, biotechnological application

## Abstract

The microbial cell membrane is affected by physicochemical parameters, such as temperature and pH, but also by the specific growth rate of the host organism. Homeoviscous adaption describes the process of maintaining membrane fluidity and permeability throughout these environmental changes. Archaea, and thereby, *Sulfolobus* spp. exhibit a unique lipid composition of ether lipids, which are altered in regard to the ratio of diether to tetraether lipids, number of cyclopentane rings and type of head groups, as a coping mechanism against environmental changes. The main biotechnological application of the membrane lipids of *Sulfolobus* spp. are so called archaeosomes. Archaeosomes are liposomes which are fully or partly generated from archaeal lipids and harbor the potential to be used as drug delivery systems for vaccines, proteins, peptides and nucleic acids. This review summarizes the influence of environmental parameters on the cell membrane of *Sulfolobus* spp. and the biotechnological applications of their membrane lipids.

## 1. Introduction

Membranes are an important barrier between the cell and extracellular space and thus act as a physical barrier as well as a gatekeeper. Their semi-permeable function is vital for the cell’s survival. Hence, maintaining membrane permeability and fluidity throughout changes in environmental parameters like temperature, pH, pressure, mechanical stress, energy availability and others, is a key step for ensuring optimal membrane function and thus viability of the cell. Cells can achieve this by altering the lipid composition of their membranes [1]. This adaptive response, termed homeoviscous adaption, was first proposed by Sinensky in 1974. He investigated the changes in membrane compositions of *Escherichia coli*, in response to increasing growth temperatures [2]. In prokaryotic organisms, it was shown that non-lamellar-forming membrane lipids are essential for specific membrane functions. This non-bilayer structure formation is influenced by growth conditions, such as the uptake of fatty acids and temperature. The phase transition temperature of the membrane lipids is modified by acyl chains and polar head groups, and therefore either a lamellar or non-lamellar phase constituted [3,4]. A specific ratio between the lipids forming bilayer and non-bilayer was proposed to be maintained in *E. coli* [5,6]. This phenomenon of organisms altering their membrane lipid compositions in response to changes in their environmental conditions has been observed in all three domains of life. Adjustments in unsaturation levels, hydrocarbon chain-length or polar head group compositions have been observed as an adaption mechanism to changes in temperature, pressure, pH and growth phase [7,8]. This process conserves physiological homeostasis. In contrast to Bacteria and Eukarya, Archaea harbor fundamentally different membrane lipids comprised of hydrocarbon chains, linked by ether bonds rather than ester bonds [9]. In general, Archaea can thrive in extreme environments, such as in high/low temperature, high/low pH values, high pressure and high salinity, among others [10], abilities that are rooted in the unique archaeal membrane and lipid structure. Many studies demonstrated the modifications of these archaeal lipids following deviations of their typical growth conditions. As a result of these alterations, the membrane becomes either more rigid and tightly packed, or vice versa. The membrane barrier is especially important in the archaeal genus *Sulfolobus*, as these species maintain an almost neutral cytoplasmic pH of 6.5, albeit growing in an acidic environment with a pH of 2.0 to 4.0 [11]. Protons are continuously pumped out, thus creating a chemiosmotic gradient, which in return, rejects positively charged protons [12].

Archaeosomes, liposomes made of archaeal membrane lipids, are a prosperous approach for oral drug delivery. Their stellar durability towards temperature and pH offers a superior alternative to liposomes made out of phospholipids. As growth conditions and environmental factors affect the membrane compositions of Archaea and thus the characteristics of their lipids, the very same approach can be used to steer the properties of the then assembled archaeosomes, in order to tailor their properties for specific applications [13,14].

In this review, an overview of the membrane’s homeoviscous adaption of Sulfolobales is given, as well as the current prosperous biotechnological application in the form of archaeosomes.

## 2. Cell Membrane and Lipids of *Sulfolobus* Spp.

The thermoacidophilic genus *Sulfolobus* belongs to the phylum Crenarchaeota and is a promising player for biotechnology [15], since it harbors a couple of valuable products, such as extremozymes [16], trehalose [17], archaeocins [18] and lipids for producing archaeosomes [13]. Genetic tools for this genus have rapidly advanced in recent years [19], generating new possibilities in basic science and for biotechnological applications alike. The cultivation conditions are preferably at around 80 °C and pH 3 [20]. *Sulfolobus* species are able to grow aerobically and can be readily cultivated on a laboratory scale. These organisms became a model organism for Crenarchaeota and for adaption processes to extreme environments [21,22,23,24,25,26]. *Sulfolobus* species were found in solfataric fields all over the world [27].

A major drawback of cultivating this organism was the lack of a defined cultivation medium. Until 2019, the “Brock Medium” served as the prevalent used cultivation medium. However, recently, a defined cultivation medium, the so-called “Vienna Defined Medium” or “VD Medium”, was developed. By using this, typical shortcomings of complex media like batch-to-batch variability, as well as the occurrence of inhibiting compounds, can be eliminated, while average specific growth rate and final cell density of the model Archaeon *Sulfolobus acidocaldarius* match the Brock medium [28].

### 2.1. Cell Membrane Structure

Already, in 1973, the cell wall of *S. acidocaldarius* was isolated and investigated for its compounds and biochemical characteristics. These fundamental experiments showed the high robustness against the treatment with enzymes and reagents; in particular, ethylenediamine tetraacetic acid (EDTA), sodium dodecyl sulfate (SDS), dimethyl sulfoxide (DMSO) and Triton X-100. The absence of peptidoglycan in the membrane distinguishes Archaea from Bacteria [29].

**Figure 1:**
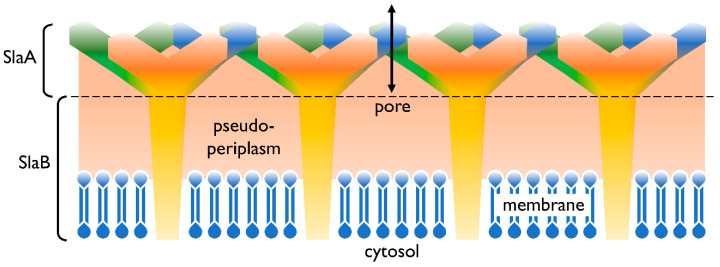
Cell envelope profile of *Sulfolobus* spp. The semi-permeable surface (S-)layer is comprised of the proteins SlaA (sheath) and SlaB (shaft). Both SlaA and SlaB are protein trimers and the contact area of these trimers is indicated with a dashed line. The salmon colored area represents the pseudoperiplasmic space. In *Sulfolobus* spp., the membrane is predominantly comprised of membrane spanning tetraether lipids [25,31].

In addition to the cell membrane, the cell envelope of *Sulfolobus* spp. consists of a proteinaceous layer called the surface (S-)layer. In *Sulfolobus* spp., the proteins SlaA and SlaB [30] were identified as the components of this layer, in controversy to many other Archaea’s S-layers consisting only of one protein. In Figure 1 a graphical illustration of the cell envelope is shown. SlaA serves as the sheath, whereas the SlaB protein makes up the shaft [30] and anchors the S-layer in the cytoplasmic membrane. The space in between, spanned by the proteins of the S-layer, represents the pseudoperiplasmic space [25].

**Figure 2:**
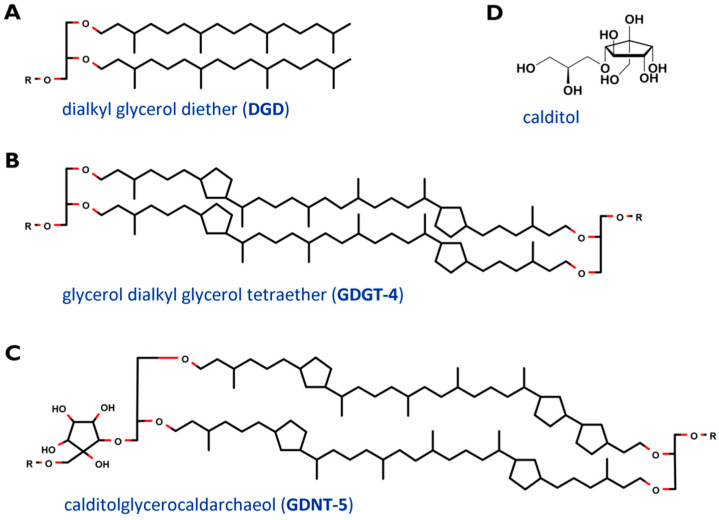
Core structure of major membrane lipids in *Sulfolobus* spp. (**A**) dialkyl glycerol diether (DGD); (**B**) glycerol dialkyl glycerol tetraether (GDGT); (**C**) glycerol dialkylnonitol tetraether (GDNT). R stands for polar head groups like (poly-) hexoses (Hex), inositolphosphate (IP), sulfonated trihexose (sulfono-Hex3), or can simply represent a single H- atom. The tetraether core structures are exemplarily depicted with 4 (GDGT-4) and 5 (GDNT-5) cyclopentane rings. (**D**) revised structure of the head group calditol, according to [35].

The cell membrane, which encloses the cytoplasm, is composed of phospho- and glycolipids. A further crucial difference between archaeal and bacterial membrane lipids is the presence of ether bonds in the former instead of ester linkages. In most Archaea, a lipid bilayer comprised of glycerol diether lipids construct the cell membrane [25]. The core archaeal lipids are monomeric dialkyl glycerol diethers (commonly called archaeol or DGD). Their hydrophobic core of C_20_ isoprenoids is linked to the glycerol backbone at the *sn*-2,3 positions (Figure 2A). Two diether moieties can form glycerol dialkyl glycerol tetraether (called caldarchaeol or GDGT), by head to head linking (Figure 2B). The hydrophobic core is then comprised of C_40_ archaeal isoprenoids. Diether lipids form a bilayer in contrast to the monolayer formed by tetraether lipids. Both diether and tetraether structures exhibit variations in their polar head groups, cyclization and length of hydrophobic core (the latter is true for the archaeol of certain Archaea only). In *Sulfolobus* spp. the constituents of the cell membranes are predominantly tetraether lipids arranged as a monolayer. Furthermore, Sulfolobales’ membranes also contain calditol linked to the *sn*-1 position of the glycerol (Figure 2C and D). This is one of the major variants of caldarchaeol, and, based on early, inaccurate structure elucidation, is also known under the deceptive name glycerol dialkylnonitol tetraether (GDNT) [32,33,34,35,36].

Table 1 gives an overview of the membrane lipid composition of the order Sulfolobales, in comparison to other well studied archaeal members. The halophilic membrane of *Natronococcus* is comprised of a mixture of C_20-20_ diether lipids (lipids harboring two alkyl chains in the lipid core, with 20 carbon atoms per chain) and C_20-25_ diether lipids (20 and 25 carbon atoms) [37], while *Sulfolobales* mainly consist of C_40-40_ tetraether lipids and partly of C_20-20_ diether lipids [9]. Methanogens like the *Methanobacterium* contain C_20-20_ diether lipids, as well as C_40-40_ tetraether lipids in varying ratios in their membrane [38]. GDNTs have only been found within the Crenarchaeota phylum so far [39].

### 2.2. Biosynthesis of Archaeal Membrane Lipids

**Figure 3:**
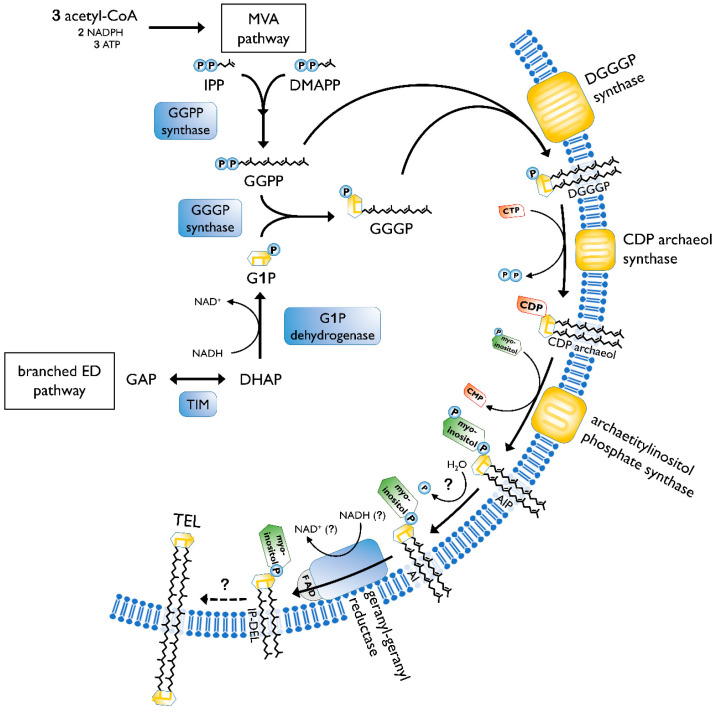
Pathway of archaeal lipid biosynthesis. MVA, mevalonate pathway; IPP, isopentyl pyrophosphate; DMAPP, dimethylallyl pyrophosphate; GGPP, geranylgeranyl diphosphate; ED pathway, Entner–Doudoroff pathway; GAP, glyceraldehyde 3-phosphate; TIM, triosephosphate isomerase; DHAP, dihydroxyacetone phosphate; G1P, glycerol-1-phosphate; GGGP, geranylgeranylglyceryl phosphate; DGGGP, 2,3-*O*-geranylgeranylglyceryl diphosphate; CTP, cytidine triphosphate; CMP, cytidine monophosphate; CDP, cytidine diphosphate; AIP archaetidylinositol phosphate; AI, archaetidylinositol; IP-DEL inositolphosphate diether lipid; TEL, tetraether lipid; P, phosphate; PP, pyrophosphate. A description of the biosynthesis steps is given in the text. Figure based on [50,54].

The biosynthesis of membrane lipids is different between the tree domains of life, as well as within one domain. In general, archaeal membrane lipids are formed by multiple consecutive enzymatic steps (Figure 3), starting with the synthesis of the isoprenoid building blocks isopentyl pyrophosphate (IPP) and dimethylallyl pyrophosphate (DMAPP). This is done via the MEP/DOXP pathway, the classical mevalonate (MVA) or the alternate MVA pathway. The alternate MVA pathway is most common in Archaea, with the exception of Sulfolobales that utilize the classical MVA pathway [50]. According to Boucher et al., *Sulfolobus* acquired the enzymes of the classical MVA pathway through lateral gene transfer from Eukaryotes [51]. Furthermore, geranyl diphosphate (GPP) is formed through condensation reactions of DMAPP and IPP. The carbon 10 compound is then condensed with several IPP molecules, to form farnesyl (C15), geranylgeranyl (C20) or farnesylgeranyl (C25) diphosphate [52]. For the formation of the characteristic ether bonded geranylgeranylglyceryl diphosphate (GGGP), geranylgeranyl diphosphate (GGPP) of the pathway described before is needed, as well as glycerol-1-phosphate. The latter is formed through the reduction of dihydroxyacetone phosphate (DHAP), utilizing NADH. The diether DGGGP (2,3-*O*-geranylgeranylglyceryl diphosphate) is formed by GGGP and GGPP. With this step, the core lipid is formed and is activated by cytidine triphosphate (CTP) in the following. This is done by the CDP archaeol synthase [50,53].

Next, the reactive cytidine diphosphate (CDP) head group is replaced by polar head groups, such as serine, ethanolamin, glycerol or *myo*-inositol. Then, geranylgeranyl reductases catalyze the hydrogenation of the unsaturated DGGGP and hence completing the structure of the archaeol [50]. It is still not clear at which step the saturation occurs. However, Koga and Morii (2007) proposed that it took place after the addition of the polar head group [52].

The caldarchaeol, which represents the characteristic tetraether lipid structure, is formed from two archaeols undergoing a head to head condensation. This was proven by labeling experiments in *Thermoplasma acidophilum* [50]. Until now, the enzyme catalyzing the tetraether bond has not been identified. Additionally, the biosynthesis of cyclopentane rings is also only partially known. A hypothesis about tetraether and cyclopentane ring formation was offered by Villanueva et al. They suggested a “multiple-key, multiple-lock” mechanism and proposed that the cyclopentane rings are being formed before the tetraethers configuration. The coupling of two diethers is hypothetically realized by phytoene synthase, which has been elucidated in the archaeal genome. However, this theory still has to be verified by experiments [55]. A strong argument against this hypothesis is that the ring index (RI) quickly changes upon changed environmental conditions [46]. Following the hypothesis from Villanueva, a change in the ring number would require a complete cycle of lipid degradation and synthesis of the new lipids with different ring numbers. The findings by Zeng et al. [56] of an ordered process of cyclization counters the proposition that the rings are already existent in the isoprenoids when added to the glycerol backbone. It confirms a common hypothesis that the cyclization occurs within the GDGT chain. Albeit, it is still not certain if the saturation of the chain, as well as the different head groups, plays a role in the ring formation process. Two genes, Saci_1585 and Saci_0240, were determined to be coding for GDGT ring synthases (Grs). Both respective proteins, GrsA and GrsB, belong to the S-adenosylmethionine (SAM) protein family [56]. Members of the same family are also responsible for the formation of the calditol moiety [39]. Hence, both incorporations require a free radical mechanism. GrsA is responsible for the introduction of rings at the C_7_ position of the core GDGT lipid. This has to be done prior to the cyclization of the C_3_ position carried out by GrsB [56]. Further, two other genes, Saci_0421 and Saci_1201, in *S. acidocaldarius* were identified for influencing the number of cyclopentane rings. If deleted, the number decreases. However, their exact function in the cyclization process has not yet been determined [57].

Additional to the typical caldarchaeol, the membranes of *Sulfolobus* also exhibit GDNT. One glycerol is replaced by a cyclopentane containing moiety, called calditol (Figure 2D). For its biosynthesis, D-glucose is converted into calditol via an “inositol-like” pathway [58]. C-C bond is formed between C_1_ and C_5_ of glucose. The possible mechanism of calditol formation includes starting the oxidation at C_4_, which resembles the formation of *myo*-inositol. Both moieties are present as polar groups in the cell membrane of Sulfolobales [59].

A connection between the lipid synthesis and homeoviscous adaption is certainly present and of crucial importance. However, on a genetic, regulatory level, this influence on the lipid biosynthesis in response to environmental changes remains an untouched field of investigation and represents a promising area for future research.

### 2.3. Effect of Cyclopentane Rings, DEL:TEL Ratio and Length of Carbon Chain on the Archaeal Membrane

The effects of cyclopentane rings on the properties of archaeal membranes is usually studied in vitro, by investigating liposomes formed of the respective diether lipids (DEL) and tetraether lipids (TEL).

TELs, the main constituents of crenarchaeal membranes, can exhibit either GDGT or GDNT as hydrophobic core. Up to eight cyclopentane rings can be incorporated within this core [60,61]. Differential scanning calorimetry and perturbation calorimetry were used to determine the effect of the incorporation of cyclopentane rings. Findings in liposomes made with extracted GDGT and GDNT lipids of *S. acidocaldarius* suggest that a higher number of cyclopentane rings in the hydrophobic core affects the membrane packing and increases the transition temperature. Albeit, the volume of the membrane is relatively constant between liposomes containing low and high amounts of cyclopentane rings [62]. In general, the compressibility, the relative change of volume in response to a change in environment conditions [63], of tetraether liposomes seems to be lower when compared to diester liposomes, meaning that TEL liposomes are less influenced by stressors such as temperature [64]. These experimental findings are backed by *in silico* results. Molecular modeling proposed that the incorporation of cyclopentane rings results in a tighter and more rigid membrane [65]. However, it was shown that the isothermal compressibility values increase with the number of cyclopentane rings in a nonlinear manner. The most tightly packed membrane was not observed at the highest number of rings, but at the optimal growth temperature of the organism [62]. These results indicate that cyclopentane rings might not be the only aspect affecting the rigidity and packing of archaeal membranes [62,65,66].

Diether lipids, like diester lipids, form bilayers, while tetraether lipids can span the lamellar structure, and thus, form monolayers [1]. Archaeosomes constructed from total polar lipid extracts of methanogens and halophiles, mainly consisting of diether lipids, are less stable against extreme pH treatment, measured by entrapped radioactive compounds, when compared to archaeosomes created from lipids extract of *T. acidophilum*. The incorporation of TEL increases the stability towards high temperature, pH treatment and serum proteins [67]. Liposomes with a high TEL content could even endure sterilization (121 °C, 1.034 bar, 15 min). There is a clear relationship between the leakage rate of a fluorescent dye and TEL presence in the membrane. Albeit, archaeosomes containing TEL or DEL can endure enzymatic treatment with pancreatic lipase [68,69]. In general, the increase of TEL content in the membrane aids the flux regulation of solutes and protons [70].

The chain-length variation of the hydrophobic lipid core is typical for Bacteria and Eukaryotes as a response to environmental stress [71]. Hereby, the difference between altering the length of hydrocarbon chains and changing from DEL to TEL has to be distinguished. While switching from diether to tetraether is a process in which two diethers are conjoined together, Bacteria alter their length of fatty acid chains; e.g., switching from C_16_ to C_18_ [72]. Membranes of alkaliphilic Archaea are composed of a mixture of C_20-20_ and C_20-25_ diether lipids, resulting in higher permeability and fluidity at low temperature. However, establishing a connection between this ratio and the framework homeoviscous adaption has not been possible yet [70,73].

In addition to high temperature adaption, methanogens, like *Methanococcoides burtonii*, can adapt to low temperature by unsaturation, forming double bonds in the hydrocarbon chain [74], a feature that appears to be absent entirely in Sulfolobales.

### 2.4. Membrane Regulators

Three primary functions have been attributed to biological membranes. First, they prevent the entrance of toxic substances into the cell. Secondly, they provide a framework for the channels and receptors responsible for the inward and outward transport of nutrients, ions and waste products. Thirdly, they provide separate environments between organelles. Membrane regulators, such as sterols, have the ability to regulate the membrane dynamics in Bacteria and Eukarya. They are known to protect the membrane from amphipathic toxins and stabilize its structure [75].

In a current review, Salvador-Castell et al. suggested possible members of archaeal membrane regulators. Polyterpenes, like carotenoids, polyprenols, quinones and apolar polyisoprenoids are likely to function as those, and help the cell membrane to adapt to physiological and physicochemical stressors. Sulfolobales feature all four of the proposed membrane regulators. As for carotenoids, β-carotene and zeaxanthin have been shown to be present in Sulfolobales. In Bacteria and Eukaryotes this said polyterpene has an antioxidant effect and intervenes with the membrane’s physicochemical properties. However, the function in Archaea is still unknown, together with the insertion process into the monolayer membrane of *Sulfolobus* spp. Polyprenols are involved in membrane protein glycosylation and protect cells against free radicals. Its length of side chains and number of unsaturations in polyphenyl-alcohol were associated with environmental conditions such as temperature. Although quinones have not been found in all Archaea, sulfolobusquinones are present in Sulfolobales. Besides its involvement in the transfer of electrons and protons in the respiratory chain, this potential archaeal membrane regulator is engaged in membrane modulation and stabilization. In *Saccharolobus solfataricus* (previously *Sulfolobus solfataricus*), the quinone side chains are affected by oxygen availability and carbon source. Apolar polyisoprenoids’ function is still being investigated. Longer apolar isoprenoids are present in hyperthermophilic *Thermococcales*, as well as extreme halophiles such as *Haloferacales* and *Halobacteriales*, while thermoacidphilic Sulfolobales synthesize mainly short ones. This can be due to the fact that long-chain polyisoprenoids are correlated to bilayer and not monolayer, which *Sulfolobus* membrane is mainly comprised of due to the high tetraether lipid content. In the bilayer, this polyterpene acts as a membrane stabilizer towards stress factors [76].

## 3. Homeoviscous Adaption

Homeoviscous adaption has been studied in several Sulfolobales species, especially in the most commonly used *S. solfataricus*, *Sulfolobus islandicus* and *S. acidocaldarius*. The focus of the research is on (1) the DEL:TEL ratio; (2) the amount of cyclopentane rings; and (3) the polar head groups. The amount of cyclopentane rings (= cyclization number) is commonly described via an averaged parameter called ring index (RI) [77]. RI is the weighted average number of cyclopentane rings in the entire TEL pool of the membrane lipids (Equation (1)). The ring number of individual lipid species is indicated with a number ranging from 0-8 added after the lipid class (e.g., GDGT-4). Polar head groups are connected at the glycerol’s *sn*-1 position and are found to be hexoses (Hex), inositolphosphate (IP) and sulfonated trihexose (sulfono-Hex3) [66].

Equation (1) shows the ring index (RI) formula. n represents the number of cyclopentane rings and GDGT-n.

(1) $$RI=\frac{\sum_{n=0}^{8}n\times \left[\%GDGT\left(n\right)\right]}{100}$$

Table 2 gives an overview of the correlations between the alterations in the membrane lipid composition and the applied growth conditions according to present knowledge. The connections between the modifications and the changes in temperature, pH and growth rate are discussed further in the next sections.

### 3.1. Temperature

*Sulfolobus* spp. are thermophilic organisms with an optimal cultivation temperature of about 80 °C. In order to ensure or control membrane fluidity and permeability at these harsh cultivation conditions, Archaea developed adaption strategies. The archaeal cell membrane can be distinguished from the bacterial due to characteristic ether bonds in the membrane lipids. Numerous thermophilic Archaea also combine the presence of tetraether lipids in their membrane. However, *Methanopyrus kandleri*, a hyperthermophilic species, growing at 90 °C, only contains diether lipids in its membrane, contradicting the proposal that GDGT is needed for survival at high temperatures. However, no further exceptions like *M. kandleri* have been discovered yet. Due to the low phase-transition temperature of archaeal membranes, they remain in liquid crystalline phase from 0 °C to 100 °C [80,81,82].

Already, in 1980, a connection between temperature and ether lipid composition was observed in *S. solfataricus*, then known as *Caldariella acidophila*. In this early research, a trend of increased incorporation of cyclopentane rings in TELs with rising cultivation temperatures was observed. The RI increased from around 2 to 2.5, in respect to a temperature change from 75 °C to 89 °C. Interestingly, GDNT was stronger affected by cyclization processes than GDGT. IP-GDGTs were minimally affected by temperature with regards to the average cyclization [46]. For two other species, *S. islandicus* and *Sulfolobus tokodaii*, the temperature dependent lipid composition was also investigated. In both species the lipid species differed for the applied growth conditions. Albeit, no clear connection between temperature and the presence of specific lipid classes varying in the polar headgroups was observed. Generally, more cyclopentane rings embedded in the GDGT lipid occurred, with increasing temperature in both species. GDNT could not be detected due to a lipid standard issue. Hexose-GDGT-inositolphosphate’s RI increased from 3.7 to 4.5 in *S. islandicus* and from 4.7 to 5.9 in *S. tokodaii*, as a result of a 10 °C increase. In contrast to the above-mentioned study with *S. solfataricus*, the same trend was observed in IP-GDGT, with a similar RI shift. Sulfono-Hex3-GDGT-IP cyclization showed the least changes. The RI in *S. tokodaii* is generally higher than in *S. islandicus,* which can also be a result of the lower cultivation pH of 3 rather than 4, respectively [42]. Nontheless, apparently there are significant difference in the average cyclization number in Sulfolobales (*S. solfataricus*: RI = 1.74 [46], *S. acidocaldarius:* RI = 4.6 [43]). Different DEL:TEL ratios were observed in *S. tokodaii* and *S. islandicus*, in respect to the applied variations of temperatures. However, no clear trend was detected [42], as it is found in the hyperthermophilic archaeon *Thermococcus* spp. Both *T. kodakaraensis* and *T. barophilus* have been shown to change the ratio of DEL to TEL in their membrane as an adaption mechanism to increased cultivation temperature [83,84]. This overall trend of increase in the number of cyclopentane moieties with increasing temperature can also be observed in other Creanarcheota, such as *Acidilobus sulfurireducens*, a species also isolated in the Yellowstone National Park, and in Crenarchaeota found in marine sediments [85,86]. Simulations of tetraether lipid membranes of *S. acidocaldarius* revealed physical properties in combination with varying cyclopentane rings and temperature. Membranes solely comprised of GDNT-4 formed more flexible structures at 77 °C when compared to GDNT-0 membranes. Incorporating rings into their hydrophobic core of GDNT lipids enhances the tensile strength of the membrane [87].

To sum up, a positive trend between temperature and RI was observed. Increasing growth temperature increases the average cyclization in the lipid classes [42,46]. Changes in the DEL:TEL ratio with differing temperature were pointed out, however, no clear trend was seen. Same was observed regarding the varying polar headgroups. On the other hand, the effect of temperature on the DEL:TEL ratio was only investigated by Jensen et al. [42]

### 3.2. pH

Homeoviscous adaption also occurs in response to pH changes. Acidophilic Archaea have adapted to life at low pH while maintaining a near neutral cytoplasmic pH. This is achieved by several mechanisms. The intracellular transmembrane potential is positive, thus repelling protons. Further, an overflow of protons is prevented by efflux systems. The membrane, in general, exhibits low proton permeability. Archaeal membranes comprised of monolayer forming TELs are an example of highly impermeable membranes [80,88,89,90]. As already mentioned in the previous section, pH potentially has an effect on the average cyclization number. Boyd et al. showed that by decreasing the cultivation pH, the GDGT cyclization was increased in the crenarchaeon *A. sulfurireducens*. [86]. This is contradicted by experiments in *Thermoplasma acidophilum*, which indicate a positive correlation between ring index and pH [91]. These results suggest additional factors to be involved in the pH adaption process, such as usages of different polar head groups. Several studies showed that the sugar moieties are faced outwards and their hydroxyl groups presumably aid the repulsion of protons on the cell surface [88,92].

McCarthy et al. applied experimental evolution to investigate the thermoacidophily of *S. acidocaldarius*. Over a three-year period employing adaptive evolution, a super-acid-resistant Crenarchaeota (SARC) strain with possible culture conditions of pH 0.8 and 80 °C was established. Transcriptomics showed upregulation in S-adenosyl methionine (SAM) proteins, which is required for calditol synthesis, and further increased the regulation of the membrane lipid biogenesis. They argue that, according to the thin layer chromatography (TLC) results, and after using lipid-specific dyes, no significant difference in the total lipids and glycolipids was observed. However, upon close inspection of the published images of the TLC plates, it appears that a difference in the total lipid compositions between SARC cultivated at pH 3 and pH 1 can be seen [93]. Sulfolobales contain GDNT in their membrane, in contrast to many other Archaea. Only *Acidianus* and *Metallosphera*, both Crenarchaeota, have been shown to incorporate GDNT, as well, in their cell membrane. SAM protein is required for calditol synthesis, and deletion of the gene locus encoding for this protein showed reduced acid tolerance of the mutant strain. The calditol synthase (*cds*) mutant was still able to produce glycosylated GDGTs, but was not able to survive at pH 1.6. Growth at pH 3.5 was not inhibited when compared to the parental strain, suggesting that the remaining glycolipids suffice as a protection. A hypothesis is that the hexose head group in the *cds* mutant cells get removed due to the exposure to low pH. This process then results in an increased membrane permeability. Hence, the stronger calditol-linked tetraethers are needed for a higher acid tolerance [39]. Moreover, the presence of potassium ions in the supplied media are supposedly needed for acid tolerance in *S. acidocaldarius*. This leads to the proposal that other factors, such as media composition, also play a role in the coping mechanisms of Sulfolobales towards pH stress [94]. In addition, the presence of proteins, in particular heat shock protein 20 (Hsp20), can prevent stress-induced protein aggregation and membrane destabilization in archaeosomes [95]. Secretory vesicles and thus archaeosomes are unable to perform pH homeostasis, necessary for maintaining a neutral intrinsic pH. This results in a pH of 2 to 3 inside theses vesicles. It is unclear how long it takes for the pH to decline in these vesicles, but according to research conducted with archaeosomes generated from tetraether lipids derived from *T. acidophilum*, the presence of TEL increases the pH resistance measured by the leakage of fluorescent or radioactive entrapped compounds when compared to archaeosomes comprised of mainly DELs [67]. Roy et al. observed that Hsp20, found in the vesicles, protected the protein lactate dehydrogenase from aggregation at a pH of 3. The small heat shock proteins (sHsps) interaction with membrane lipids occurs via hydrophobic interaction. When incubated with archaeosomes, interaction with the membrane lipids was observed. Atomic force microscopy (AFM) images confirmed the membrane stabilization ability of Hsp20, after treating the vesicles at 65 °C for 30 min. By the addition of Hsp20, liposomes remained intact. Also, the stability of archaeosomes that were exposed to increased temperatures was shown to be enhanced by Hsp20. This was determined by measuring the leakage of the fluorescent molecule 1,6-diphenylhexatriene (DPH) in the presence and absence of Hsp20. According to these findings, the presence of heat shock proteins in archaeosomes of *S. acidocaldarius* has an important impact on acid tolerance, as well as temperature related effects in regards to the membrane characterization and packed protein [95].

Modifications in the cyclization pattern and in the DEL:TEL ratio in dependence of pH were not examined in Sulfolobales. However, acid tolerance could be linked to the upregulation of SAM proteins, required for calditol synthesis, and generally membrane lipid biogenesis [93]. Furthermore, the presence of the headgroup calditol was also shown to be necessary for survival at low pH [39].

### 3.3. Growth Rate

Recent studies by Jensen et al. [42], Bischof et al. [79], Zhou et al. [78] and Quehenberger et al. [43] confirm that growth phase/rate also have an effect on the lipid composition of Sulfolobales. Jensen et al. observed that the lipid compositions of *S. islandicus* and *S. tokodaii* not only changed with the cultivation temperature, but also with the growth phase. A distinction was drawn between lag, exponential and stationary phase. Although the presence of the 3 investigated lipid classes changed with the growth phases, it was not possible to identify a clear trend between the occurrence of each lipid class and the different growth phase and no apparent connection was found between the changes in growth phase and the increasing and decreasing DEL:TEL ratios. The average cyclization of the total lipid extracts underwent a dip from lag phase to exponential phase, followed by an increase in the stationary phase. A possible explanation is the overwhelmed machinery of cyclization incorporation or synthesis at such high growth rates [42].

A different experimental approach was taken by Bischof et al. *S. acidocaldarius* was deprived of its nutrients and thus the growth rate was reduced. The survival rate and the cytoplasmic pH, as well as the membrane composition, were monitored. A longer depletion led to a lower viability and a decrease in the cytoplasmic pH. Upon nutrient depletion, the average cyclization number stayed the same with an RI of 3.4, whereas when cultivated with nutrient rich medium the ring index slightly decreases. The amount of GDGT with 3 cyclopentane rings clearly increased with time when the cells were not nutrient deprived, while the same lipid class decreased in starved cells. This probably is part of the stress response, making the membrane less permeable by tighter packing. The following decrease in RI in the cells supplied with nutrient rich medium could be due to the fact that the cells were still in the exponential phase at the time point that the different conditions were applied. Harvesting and subsequent transfer into nutrient rich and depleted medium was performed at an OD_600_ of 0.4 to 0.5, which might suggest that, at this time point, the cells were still in the exponential phase and the previously described drop in average cyclization still took place. A slight decrease in the DEL amount upon nutrient depletion was observed, as shown in their Supplementary Material [79].

Zhou et al. conducted continuous cultivations and regulated the growth rate via the feed addition while a constant temperature, pH and aeration rate was given [78]. Slower growth rates increased the cyclization, which is in conformity with the experiments conducted with the nutrient depleted medium by Bischof et al. [79] The RI changed from 2.0 in the highest applied growth rate of µ = 0.069 h^−1^ to 3.2 when a slow growth of µ = 0.01 h^−1^ was set. The amount of GDGT-4 to 6 increased with the decreasing growth rate. This confirms the implication that the availability of nutrients decreases the membrane packing and thus increases the membrane permeability towards ions, and vice versa. The tighter membrane packing and thus decreased membrane permeability at lower growth rates is important for reducing proton leakage. Hence, the pH homeostasis is maintained, which is vital for this thermoacidophilic archaeon. The amount of DEL and thus the DEL:TEL ratio was not discussed in this publication [78].

Similar results were obtained by Quehenberger et al., in a continuous stirred tank reactor with 2.0 L culture volume. Two different growth rates, µ of 0.011 and 0.035 h^−1^, were employed by controlling the feed rate. At the lower growth rate, the RI increased to 5.1 when compared to the RI of 4.6 in the higher growth rate [43]. This negative correlation is in accordance with the findings of the previously mentioned research by Zhou et al. [78] and Bischof et al. [79] In contrast to the RI, the DEL amount increased with slower growth rate, resulting in a shift from a DEL:TEL ratio of 1:5.5 (µ = 0.035 h^−1^) to 1:3 (µ = 0.011 h^−1^) [43]. Similar ratios were observed by Jensen et al. [42] in *S. tokodaii* and *S. islandicus*, although a clear trend could not be identified. Bischof et al. [79] reported rather low DEL amounts of 0.1–0.2% in *S. acidocaldarius*, making the calculation of a DEL:TEL ratio unfeasible. This seems to be an underestimation of the archaeol content.

Overall, increased growth rate results in a lower RI [43,78,79]. Regarding the DEL:TEL ratio, a negative correlation [43] in response to the growth rate was observed, as well as an unclear trend [42]. No clear observation was drawn regarding the modification of the head groups [42,43].

## 4. Extraction and Lipid Analysis

In a lot of cases, contradictory results can be a consequence of different extraction or detection methods. Exemplarily, it has been shown that the lack of a lipid class in the membrane structure was due to the extraction method, rather than an actual observation [96,97].

The organic extraction of lipids from biological tissue samples was first described by Folch et al. in 1957. It consists of chloroform/methanol/water, in a ratio of 8:4:3 (*v*/*v*/*v*). The idea was that after homogenization in chloroform and methanol mixture, water is added and thus, forms a new aqueous phase. The polar upper phase consisting of methanol and water then contains the hydrophilic compounds, whereas the lower chloroform phase holds the lipids. The recovery of phospholipids and neutral lipids is rather efficient with this method [98,99]. Bligh and Dyer modified this method to a volumetric ratio of 2:2:1.8 of chloroform/methanol/water, thereby reducing the amounts of chloroform and methanol. In general, the extraction procedure is the same as by Folch et al. However, other groups already showed that this system has limitations, especially towards neutral lipids. Modification to the protocol has been already suggested by Bligh and Dyer themselves. However, their proposed re-extraction steps were not commonly applied [100,101]. Other alterations included the addition of HCl for the better recovery of total fatty acids and polyunsaturated fatty acids [102] and acetic acid for oxidized fatty acids [103]. However, the application of acid hydrolysis removes the polar headgroup of the GDGTs, and consequently, it is only possible to recover core GDGTs [104]. The quantitative recovery of each lipid class is influenced by many variables. Especially, the choice of extraction solvent is important, as it determines the lipid class that is extracted. Non-polar solvents, such as hexane, extract neutral lipids. The polar methanol is used to extract charged phospholipids. More polar glycolipids need a mixture, for example, of methanol and acetone. Additionally, the pH and ion strength of the solvent mixture can also have an impact on the recoveries. The recoveries are greatly dependent on the extraction efficiencies and determining the extraction recovery is unfortunately difficult [105].

The mentioned articles regarding the observation of homeoviscous adaption in Sulfolobales mainly used methanol and chloroform in varying ratios as extraction solvents [42,43,91,93]. Chen et al. and Zeng et al. used methanol in combination with dichloromethane, which has similar physical and chemical properties as chloroform, but exhibits less carcinogenic and hazardous characteristics, and was shown to be an effective alternative [39,79,106]. Zhou et al. used methyl-tert-butyl-ether (MTBE) and methanol in a ratio of 1:1 (*v*/*v*). MTBE’s low density causes the formation of the upper phase, including the extracted lipids, facilitating their collection when compared to the methanol/chloroform solvent system [78,107]. As Zhou et al. and Quehenberger et al. applied similar cultivation strategies in *S. acidocaldarius* and subsequently determined the RI number, which seemed to be generally lower in the first publication, one might wonder if the difference can be attributed to the used solvents. Quehenberger et al., who used the common ratio of 2:1 of chloroform:methanol, discovered higher average cyclization numbers of around 4.6, consistent with the observations in *S. tokodaii* and *S. islandicus* by Jensen et al. [42,43,78]. These results already indicate that a uniform lipid extraction method has to be established, in order to facilitate comparison as well as draw conclusions of the results in their entirety.

An additional factor of discrepancy concerning the found lipid classes is the detection method. TLC results are sometimes difficult to interpret, and the method should therefore only be used as separation step prior to an identification. TLC separates the components of the mixture by its polarity. For the visualization of lipids, methods such as the incubation in 8-anilino-1-naphtalene or sulfuric acid and afterwards heating can be used. Advantages are rapid and cheap analysis. Limited reproducibility and the lack of making a qualitative, but only a qualitative, analysis are the major disadvantages of this method [108,109].

Quantitative analysis and exact identification of the lipid classes require mass spectrometric (MS) procedures. Analyte ionization occurs via electrospray ionization (ESI), electron impact (EI), matrix assisted laser desorption/ionization (MALDI), or by atmospheric pressure chemical ionization (APCI). ESI, MALDI and APCI are so-called soft ionization techniques, and in contrast to EI, can be used for non-volatile analytes. After analyte ionization, the mass dependent ion separation and ion detection takes place in the mass spectrometer. Different lipid classes are then identified by the mass to charge ratio (*m*/*z*). Lipid analysis can be separated into the shotgun lipidomics approach and using MS in tandem with other separation techniques such as chromatography. In shotgun lipidomics, the crude total lipid extract is directly inserted into the MS, with prior analyte ionization, but without prior separation. However, ion suppression is a major disadvantage of this shotgun procedure. A consequence of this phenomenon is that low abundant species might be missed. This drawback is partially overcome by chromatography methods. Liquid chromatography (LC) techniques can separate the lipid classes according to their carbon chain length and the presence of double bonds [110,111,112].

The accurate quantification of lipid classes requires internal standards, which have to exhibit very similar physicochemical properties as the analyte, but can be distinguished by different *m/z* values [113]. Labeled standards for archaeal lipids are not widely commercially available. Only one tetraether lipid standard is supplied by Matreya LLC (State College, PA, USA). It is a lipid extract of the main polar lipid (Hex-GDGT-PG) of *T. acidophilium*. This was used for quantification by Jensen et al. They remarked that it contains contaminating amounts of GDNT and Hex-GDNT, and therefore these lipid classes could not be quantified in their analysis [42]. Apart from that, the other discussed research articles either did not specify their used lipid standards [43,46,78], or used internal standards instead [39,79,86]. Boyd et al. used lipid extracts of *S. acidocaldarius* for the quantification of the membrane lipids in *A. sulfurireducens* [86]. Bischof et al. [39] and Zeng et al. [79] used synthesized C_46_ GDGT for the determination of GDGT abundance. The synthesis was established by Patwardhan and Thompson, and the quantification method by Huguet et al. [114,115].

The recent publications all identified their lipid classes by MS approaches [42,43,78,79]. However, the used quantification standards were either not the same, or not specified. Hence, the contradictory results could be a result of different extraction methods or quantifications methods, if not as a result of the applied cultivation conditions.

## 5. Applications

The monolayer architecture of archaeal membranes provides high mechanical strength and the lipid structure characterized by ether bonds, cyclopentane rings and saturation results in high enzymatic, chemical [116] and physical [117] stability.

Generally, these characteristics of ether lipids, like stability towards high temperature and acidic pH, make them potentially interesting for biotechnological applications. The use as vehicles for gene or drug delivery, as so called archaeosomes, has received increasing attention [118]. For this application, their temperature stability is a crucial advantage over conventional liposomes made from ester lipids, during processing and storage, whereas the stability towards low pH predestines archaeosomal formulations for the use in oral drug delivery, since they protect their cargo from degradation in the stomach. In 1990, Lo and Chang verified that polar lipid extracts of *S. acidocaldarius*, grown at ~65 °C, could be used for the generation of archaeosomes [119]. Those formed archaeosomes exhibit the low permeability of the encapsulated fluorescein, due to the tight and rigid packing [120].

The use of archaeal membrane lipids for the generation of archaeosomes and their applicability have already been reviewed in several articles [69,118,121,122]. Therefore, this chapter focuses on the latest findings regarding archaeosomes generated by lipids extracted from the *Sulfolobus* genus.

Generally, the most recent studies with archaeosomes are dominated by oral applications and are characterized by the fact that the investigated archaeosomes are constituted of a mixture between conventional lipids and TELs, e.g., the polar lipid extract of *Sulfolobus* was used to enhance the properties of conventional liposomes. Archaeosomes composed of phospholipids, cholesterol and archaeal lipids derived from *S. islandicus* showed that the vesicles could withstand in vitro exposure of bile salt measured by the loss of the entrapped marker. In contrast to the liposomes containing archaeal lipids, the conventional liposomes, composed of phosphatidylcholine, released almost the entire marker when incubated with bile salt. This serves as proof-of-concept for the possible usage of archaeosomes as an oral drug delivery system [123]. Archaeosomes were also used as carriers for insulin. They demonstrated higher stability in the gastrointestinal environment, in addition to overall lower levels of blood glucose indicated by an in vivo study, when compared to conventional liposomes [124]. Additionally, a number of in vivo studies have been carried out with archaeosomes to insulin, including applications for antibiotics, cancer, hepatitis B and D, as well as osteoporosis treatment [125,126,127,128,129].

**Figure 4:**
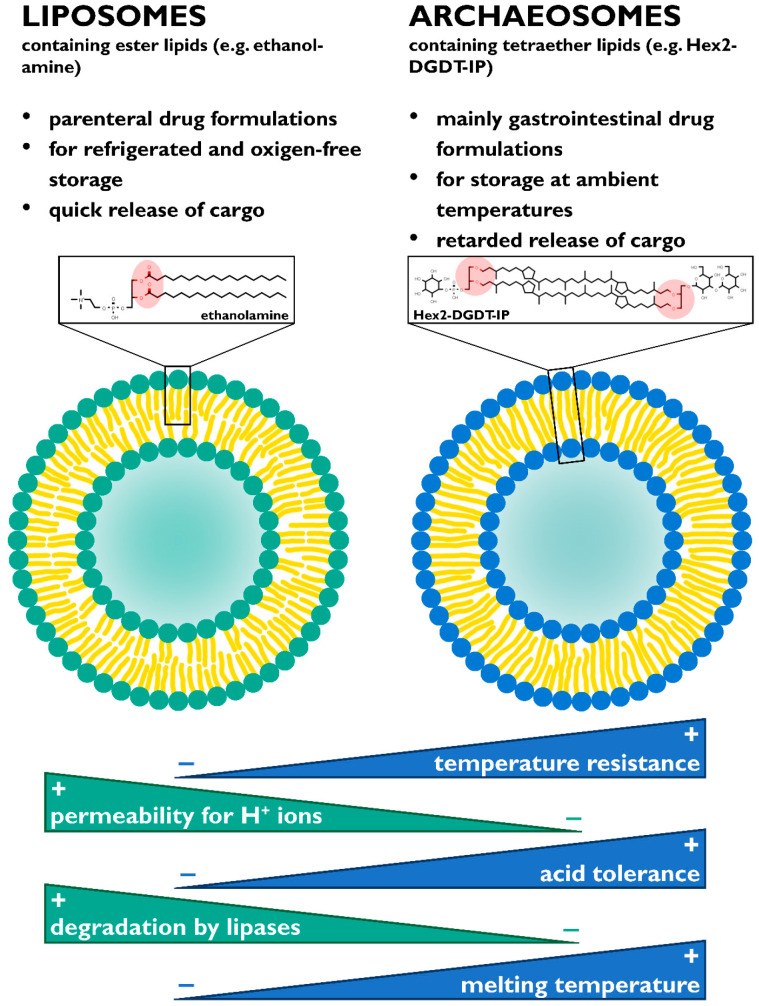
Summary of properties and applications of archaeosomes and comparison between archaeosomes and conventional liposomes.

A summary of the properties of archaeosomes in respect to the currently prevalent application of oral drug delivery in comparison with conventional liposomes is given in Figure 4.

Apart from the oral delivery of drugs, archaeosomes of *S. acidocaldarius* packed with methylene blue indicated a potential use in skin permeation treatment, as observed on rat skins. Albeit, the addition of cholesterol enhanced encapsulation, and thus, the release of the marker through the skin [130]. TELs extracted from *S. acidocaldarius* in combination with phosphocholine were used to generate a liposome for the encapsulation of a drug called Ce6 used in the photodynamic therapy for cancer treatment. Due to the TELs in the liposomes, a slower release of the drug to the cancer cell could be achieved [131]. In another study, mixing cholesterol and TEL extracted from *S. acidocaldarius* resulted in a stable vector for gene delivery used for the transfection of mammalian cells [132]. These applications, including the oral delivery of drugs, are summarized in Table 3.

In summary, archaeosomes, specifically made with lipid extracts of *Sulfolobus* genus, were found to be used for oral and intravenous drug delivery, gene delivery or as a skin permeation system. In most of the cases, the archaeal lipids were mainly used as an enhancer of the conventional lipids, due to their extraordinary characteristics. However, it was also demonstrated that the generation of “pure” archaeosomes is also feasible and these are more resistant to pH, making them act as great oral drug delivery systems.

Growth environments differing in temperature, pH and growth rate cause modifications in the lipid structure in Sulfolobales, making their membrane more or less rigid. In the same way, based on this concept of homeoviscous adaption and the connection between lipid composition and membrane properties, it should be possible to steer the characteristics of the archaeosomes and to tailor them for specific applications. Nevertheless, until now, it has not been systematically investigated whether the different growth conditions resulting in different lipid modifications, such as cyclopentane rings, have an impact on the archaeosome properties.

## 6. Conclusions

Modifications in the lipid composition as a homeoviscous adaption strategy are done to alter the membrane packing and therefore maintain membrane fluidity. A lower DEL:TEL ratio increases the membrane rigidity and generates a less permeable membrane. Subsequently, the incorporation of cyclopentane rings enhances this process even further. The membrane lipid composition of *Sulfolobus* spp. is influenced by growth conditions, such as pH, temperature and growth rate. The RI number is positively correlated with temperature, while decreasing when the growth rate increases. Both observations were reported in multiple publications. Generally, the DEL:TEL ratio was shown to change in response to temperature as well as growth rate. However, in two studies, no clear trend, and only in one case, a negative correlation in regards to varying growth rates, was observed. In terms of the presence of different polar head groups, again, the connection between temperature and growth rate remains obscure. Solely the presence of the calditol moiety was shown to increase acid tolerance. Altogether, highly cyclized GDGT could be a general response to stress in Sulfolobales. Generally, reports on the membrane lipid composition have to be critically reviewed according to the used extraction and detection methods, as these have an impact on the obtained results. A standard extraction method capable of extracting all lipid classes comprised in the archaeal membrane should be applied in all further studies. Chloroform, in combination with methanol, has been shown to achieve the best extraction results in terms of total lipid extraction efficiency. Conclusions in regard to the lipid composition upon results acquired by TLC are not adequate. Mass spectrometry, or mass spectrometry along with prior chromatography steps, are necessary for exact identification of the lipid class and its quantification. Apart from this, lipid standards for quantification have to be established, as currently only one is commercially available.

While of fundamental interest for unraveling the interactions between microorganisms with their environment, the study of archaeal lipids is also of high biotechnological importance, since lipids of Sulfolobales can be used to generate archaeosomes, which have been shown to have superior properties when compared to the conventional liposomes. Studies with entrapped insulin revealed excellent stabilization of the archaeosome in the gastrointestinal tract. Further, skin permeation as a potential application has emerged, guiding the way for the establishment of new bioproducts.

## Figures and Tables

**Table 1:** Overview membrane lipid composition of *Sulfolobales*, *Natronococcus* and *Methanobacterium*.

Membrane Lipids	*Sulfolobales*	*Natronococcus*	*Methanobacterium*	Reference
**C_20-20_ DGD**	**Σ15%**–**30%**	**Σ57%**–**77%**	**Σ12.8%**–**50%**	[40,41,42,43,44]
DGD	~5%	n.d.	**+**	[38,42,43]
IP-DGD	10%-30%	n.d.	**+**	[38,42,43]
PG	**-**	8–26%	**-**	[44]
PGP	**-**	49–51%	**-**	[44]
PGP-Me	**-**	**+**	**-**	[45]
**C_20-25_ DGD**	**-**	**Σ22%**–**44%**	**-**	[44]
PG	**-**	8%–9%	**-**	[44]
PGP	**-**	14%–35%	**-**	[44]
C_40-40_ TEL (GDNT/GDGT) *	**Σ70**–**85%**	**-**	**Σ50%**–**83% (GDGT only)**	[40,41,42,43]
TEL	7–9%	**-**	n.d.	[42,43]
Hex2-TEL	57.8%	**-**	n.d.	[42,43]
IP-TEL	3–36%	**-**	n.d.	[42,43]
Sulfono-Hex3-TEL-IP	1–11%	**-**	n.d.	[42,43]
Hex-TEL-IP	**+**	**-**	n.d.	[9]
Hex2-TEL-IP	4%–82%	-	n.d.	[42,43]
C_40-40_ GDNT	Σ 68–80%	-	-	[34,46,47,48,49]
Hex-GDNT	**+**	**-**	**-**	[46,47]
IP-GDNT	**+**	**-**	**-**	[46,47]

* Recent publications [42,43], that investigated the different head groups of TELs from the *Sulfolobus* genus (*S. acidocaldarius*, *S. islandicus* and *S. tokodaii*), did not distinguish between GDNT and GDGT, due to a lack of suitable lipid standards or for methodological reasons, since these publications relied on mass spectrometry data only. DGD, dialkyl glycerol diether; IP, inositolphosphate; PG, diphytanylglycerol analogue of phosphatidylglycerol; PGP, diphytanyl-glycerol analogue of phosphatidylglycerol-phosphate; Me, methyl group; TEL, tetraether lipid; GDNT, calditolglycerocaldarchaeol; Hex, hexose; GDGT, glycerol dialkyl glycerol tetraether; Hex2, dihexose; Sulfono-Hex3, sulfonated trihexose; +, present but not quantified; **-**, absent; n.d., no data available; Σ, sum of lipid classes with different headgroups within the sub category C_20-20_ DGD, C_20-25_ DGD, C_40-40_ TEL (GDNT and GDGT). Sub categories of lipid classes are marked as bold.

**Table 2:** Correlation between changes in temperature, pH and growth rate and the responses ring index (RI), diether to tetraether lipid ratio (DEL:TEL ratio) and headgroups in *Sulfolobus* spp.

Cultivation Conditions	Modifications		
	Ring Index (RI)	DEL:TEL Ratio	Headgroups
**Temperature**	positive [42,46]	impacted, but no distinct trend determined [42]	impacted, but no distinct trend determined
**pH**	not investigated	not investigated	calditol important for acid resistance [39]
**Growth Rate**	Negative [43,78,79]	negative [43] impacted, but no distinct trend determined [42]	impacted, but no distinct trend determined [42,43]

**Table 3:** Recently investigated applications of tetraether lipids (TELs) from Sulfolobales, in the field of drug and gene delivery.

Field of Application	Specific Use	Properties Influenced by TELs	Reference
**Oral Drug Delivery**	Delivery of insulin; antibiotic, cancer, hepatitis B and D, as well as osteoporosis treatment	Protection against drug degradation in the gastrointestinal tract	[124,125,126,127,128,129]
**Dermal Delivery**	Delivery of a methylene blue as drug model through rat skin	Improved skin permeation	[130]
**Intravenous Drug Delivery**	Photodynamic therapy for anti-cancer treatment	Increased membrane rigidity for controlled release	[131]
**Gene Delivery**	Transfection of mammalian cells with TEL containing lipid nanoparticles	Increased stability and transfection efficiency	[132]

## Abbreviations

EDTA	ethylenediamine tetraacetic acid
SDS	sodium dodecyl sulfate
DMSO	dimethyl sulfoxide
NADH	nicotinamide adenine dinucleotide hydrogen
NADPH	nicotinamide adenine dinucleotide phosphate hydrogen
MVA	mevalonate pathway
IPP	isopentyl pyrophosphate
DMAPP	dimethylallyl pyrophosphate
GPP	geranyl diphosphate
GGGP	geranylgeranylglyceryl diphosphate
DHAP	dihydroxyacetone phosphate
DGGGP	2,3-*O*-geranylgeranylglyceryl diphosphate
CTP	cytidine triphosphate
CDP	cytidine diphosphate
Grs	GDGT ring synthases
SAM	S-adenosylmethionine
DEL	diether lipid
TEL	tetraether lipid
RI	ring index, cyclization number
SARC	super-acid-resistant Crenarchaeota
TLC	thin layer chromatography
*cds*	calditol synthase gene
DPH	1,6-diphenylhexatriene
AFM	atomic force microscopy
Hsp	heat shock proteins
MTBE	methyl-tert-butyl-ether
MS	mass spectrometry
ESI	electrospray ionization
EI	electron impact
MALDI	matrix assisted laser desorption/ionization
LC	liquid chromatography
Nomenclature lipids	
DGD	archaeol, dialkyl glycerol diether
GDGT	caldarchaeol, glycerol dialkyl glycerol tetraether
GDNT	calditolglycerocaldarchaeol, glycerol dialkyl nonitoltetraether
GDGT-[0–8]	GDGT with 0 - 8 cyclopentane rings in its structure
PG	diphytanylglycerol analogue of phosphatidylglycerol
PGP	diphytanyl-glycerol analogue of phosphatidylglycerol-phosphate
Headgroups	
IP	inositolphosphate
Me	methyl group
Hex	hexose
Hex2	dihexose
Sulfono-Hex3	sulfonated trihexose
